# The global end-ranges of neck flexion and extension do not represent the maximum rotational ranges of the cervical intervertebral joints in healthy adults - an observational study

**DOI:** 10.1186/s12998-021-00376-3

**Published:** 2021-05-25

**Authors:** Victoria Andersen, Xu Wang, Mark de Zee, Lasse Riis Østergaard, Maciej Plocharski, René Lindstroem

**Affiliations:** 1 Department of Health Science and Technology, Aalborg University, 9220 Aalborg, Denmark; 2 The Second Hospital of Jilin University, Jilin University, Qianjin St. 2699, Changchun, 130021 China; 3 Sport Sciences - Performance and Technology, Department of Health Science and Technology, Aalborg University, 9220 Aalborg, Denmark

**Keywords:** Maximum motion, Range of motion, Fluoroscopy, Cervical vertebrae, Neck

## Abstract

**Background:**

In clinical diagnosis, the maximum motion of a cervical joint is thought to be found at the joint’s end-range and it is this perception that forms the basis for the interpretation of flexion/extension imaging studies.

There have however, been representative cases of joints producing their maximum motion before end-range, but this phenomenon is yet to be quantified.

**Purpose:**

To provide a quantitative assessment of the difference between maximum joint motion and joint end-range in healthy subjects. Secondarily to classify joints into type based on their motion and to assess the proportions of these joint types.

**Study design:**

This is an observational study.

**Subject sample:**

Thirty-three healthy subjects participated in the study.

**Outcome measures:**

Maximum motion, end-range motion and surplus motion (the difference between maximum motion and end-range) in degrees were extracted from each cervical joint.

**Methods:**

Thirty-three subjects performed one flexion and one extension motion excursion under video fluoroscopy. The motion excursions were divided into 10% epochs, from which maximum motion, end-range and surplus motion were extracted. Surplus motion was then assessed in quartiles and joints were classified into type according to end-range.

**Results:**

For flexion 48.9% and for extension 47.2% of joints produced maximum motion before joint end-range (type S). For flexion 45.9% and for extension 46.8% of joints produced maximum motion at joint end-range (type C). For flexion 5.2% of joints and for extension 6.1% of joints concluded their motion anti-directionally (type A).

Significant differences were found for C2/C3 (*P* = 0.000), C3/C4 (*P* = 0.001) and C4/C5 (*P* = 0.005) in flexion and C1/C2 (*P* = 0.004), C3/C4 (*P* = 0.013) and C6/C7 (*P* = 0.013) in extension when comparing the joint end- range of type C and type S.

The average pro-directional (motion in the direction of neck motion) surplus motion was 2.41° ± 2.12° with a range of (0.07° -14.23°) for flexion and 2.02° ± 1.70° with a range of (0.04°-6.97°) for extension.

**Conclusion:**

This is the first study to categorise joints by type of motion. It cannot be assumed that end-range is a demonstration of a joint’s maximum motion, as type S constituted approximately half of the joints analysed in this study.

## Introduction

Neck range of motion (ROM) is a traditional method employed for the assessment of neck motion in both clinical and scientific environments [1–5]. Neck ROM is frequently assessed as a change of head position from the forward-facing upright head position to a new position after movement of the neck. Neck ROM is assessed between the head and a lower anatomical point, commonly the chin and sternal notch [6]. Neck ROM can further be divided into the motions of joints between two cervical vertebrae. Although there are multiple intervertebral joints between two cervical vertebrae (intervertebral, facet and uncovertebral joints), the multi-joint complex will henceforward be referred to as a joint.

The largest cervical joint motion associated with neck ROM in clinical diagnosis is perceived to be found at the end of the (global) neck movement [7, 8]. This perception is used in the interpretation of flexion and extension X-rays to measure the maximum joint motion. However, neck ROM contains little information about motion between the measuring points, as the measurements are taken from static positions. Studies have demonstrated representative cases where the maximum joint motion is greater than the motion found at the joint’s end-range [9–11]. Thus, in these cases the joint position at end-range could not represent the maximum joint motion.

New studies document multiple sources of joint motion variability, demonstrating that cervical joint motion cannot be perceived to be curvilinear or uniform [9–12]. Cervical joints have been demonstrated to repeat their motion and investigations of within and between day repartitions found no significant differences in joint motion angle [13]. Wang et al. concluded that the findings of their study supported the idea that the cervical joints accurately repeat their motion. Additionally, the direction of joint motion alternates between pro-directional joint motion (movement in the direction of neck motion) and anti-directional joint motion (movement in the opposite direction to that of neck motion) during neck motion [10]. The time periods and motion contributions in degrees of pro-directional and anti-directional joint motion vary, and are scattered through healthy cervical flexion and extension. Anti-directional joint motion is frequent during neck flexion and extension. For C0-C7 ROM anti-directional motion is approximately 40% of the pro-directional motion and approximately 70% of the resultant motion [10]. These results suggest that healthy cervical joints can move further than the motion found at end-range, and that this additional surplus motion is common during joint motion and may be necessary for normal healthy cervical joint motion.

Studies of cervical joint motion have previously demonstrated cervical joints with greater joint motion before end-range than at end-range [3, 9]. Intuitively, a joint’s ability to perform surplus motion would be necessary for simultaneous motion in multiple planes, as multiplane motion would be difficult if joint structures were fully stretched by motion in just one plane.

Assessment of maximal cervical joint motion in previous studies appears to be based on the assumption that cervical joints cannot move further than joint end-range and that joint motions are linear and continuous [7, 8]. This assumption is not supported by more recent studies [9–12, 14, 15]. The aims of this study were firstly, to describe the maximum pro-directional and anti-directional joint motion in 10% epochs between the initial upright position and the end-range position, exploring the relationship between maximum joint motion and joint end-range. Secondly, to analyse the maximum surplus joint motion in quartiles, and to suggest possible subdivisions of joint motion and joint classification based on type of motion. This study proposes a joint classification of single cervical joint motion types based on end range (terminal position) and the maximum joint motion.

## Methods and materials

### Definitions of concepts

Anti-directional surplus motion refers to surplus joint motion in opposition to the primary motion direction.

End-range refers in this study to the end or terminal position of a joint motion.

Epoch: An epoch is defined as a time period representative of 10% of the total time required to complete a flexion or extension neck motion.

Maximum motion refers to the maximum joint motion in degrees measured during a video fluoroscopy motion excursion. Maximum motion can also refer to the maximum motion capacity of an individual joint.

Motion excursion: A motion task performed from point A-to point B. In the case of this study, from upright to end range cervical flexion or end-range cervical extension.

Motion type refers to a classification of a single joint’s motion during neck motion. We have defined 3 types of motion, and all are defined according to their end-range.

1. Classic (C), where the maximum motion = terminal position.
2. Surplus (S), this type is classified using pro-directional surplus motion, where maximum motion is more than the terminal position.
3. Anti-directional (A) where the terminal position is less than the start position. In this study, the start position is upright and the terminal position is end-range.

Pro-directional surplus motion refers to surplus motion found beyond end-range.

Range of motion (ROM) refers to the angular motion in degrees between the start position of the motion and the end position of the motion (end-range) – range of motion can be of an individual joint or of the neck and can be measured from static or video images.

Surplus motion refers to joint motion that occurs outside the boundaries of upright (start position) and end-range. Surplus motion is the difference, in degrees, between the upright start position or the end-range position and the maximum motion for a single joint. Surplus motion can be pro-directional or anti-directional and a single joint can produce surplus motion in both directions.

Upright refers in this study to the upright start position of neck motions.

### Subjects

Due to the increased risk of cancer posed to healthy subjects by exposure to ionizing radiation, data was extracted and re-analysed from a previous study investigating the repeatability of cervical joint motion [13]. Subjects were aged between 20 and 37 and were recruited from campus and via social media, and in accordance with the following exclusion criteria: possible pregnancy, inflammatory or neurological disorders, cervical trauma, or neck pain in the last 3 months. Subjects were paid US $22 an hour.

All participants signed informed consent forms prior to participating in the study. The study was conducted in accordance with the Helsinki declaration and ethical approval was given by the regional ethics committee (N20140004).

### Experimental procedures

Both the reproducibility of image analysis and the experimental procedures have been previously published [16]. Prior to the fluoroscopic procedure subjects were instructed to practice the flexion and extension motion excursions. The subjects were instructed to follow with their eyes a line marked on the floor, wall and ceiling in order to reduce out of plane motion. Custom glasses were worn, the attached external markers provided better visual tracking of the occiput. One complete excursion was to be performed with a smooth and even tempo and to be completed at 16 s, with 2 s at the upright and 2 s at end-range positions. Subjects practiced the timed motion before imaging began and the researcher counted out loud while the subject performed the excursion under video fluoroscopy. The motion excursions were performed while sitting with knees, hips, ankles and elbows at 90° [10, 17].

### Motion analysis

Two motion directions were analysed (flexion and extension) for 7 cervical joints in 33 subjects. The motion was analysed in 10% time epochs of total cervical motion***.*** Total joint motion and total neck ROM were obtained by calculating the sum of the motion across the 10% epochs. Maximum motion, surplus motion and end- range were extracted for each joint.

Joints were subdivided into three types according to their end-range motion: Type C, end-range motion equals maximum motion; type S, end-range motion is less than maximum motion and type A, joints with anti-directional end-range motion.

**Fig. 1:**
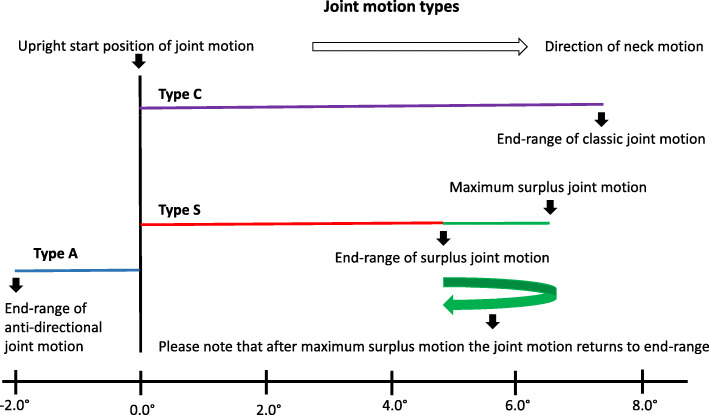
Joint motion types. Three types of cervical joint motion 1) C 2) S and 3) A. The joint motion type C illustrates the common perception of joint motion from upright along the purple line to end-range. Type C joint motion has no pro-directional surplus motion. This type of motion is documented in almost half of all cervical joints. Type S joint motion is illustrated by the red line, and beyond end-range by the green line. Almost half of the joint motion in this study was type S. The green returning arrow illustrates that the motion passes the point of end-range, before the end of neck motion, and continues to the point of maximum surplus joint motion. It then moves anti-directionally towards the end-range position. Type A joint motion terminates in the opposite direction to that of head motion. This type of joint motion is demonstrated by approximately 6% of all cervical joints and is illustrated by the blue line

**Fig. 2:**
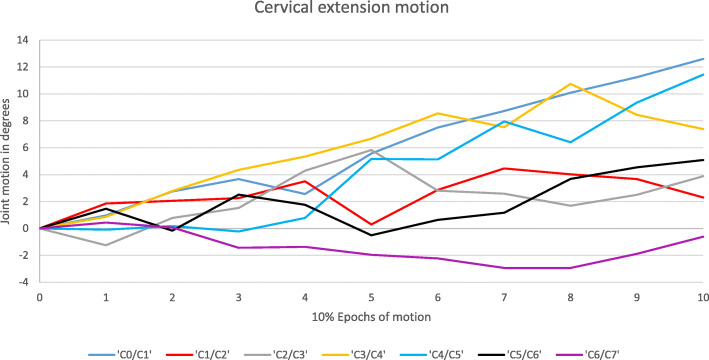
Cervical extension motion for an individual subject. C0/C1, C4/C5 and C5/C6 are type C joints, producing maximum motion at end-range. C1/C2, C2/C3 and C3/C4 is are type S joints, reaching maximum motion in the 7th, 5th and 8th epochs respectively. C6/C7 is a type A joint, terminating its motion anti-directionally

Figure 1 illustrates the three types of motion and Fig. 2 demonstrates representative cases of these motions.

### Fluoroscopic recordings

The source-to-subject difference was 76 cm, and was measured prior to exposure. The fluoroscope produces 45 KV, 208 mA, 6.0 ms X-ray pulses at 25 frames per second (Philips BV Libra, 2006, Netherland). The estimated average radiation exposure from the two fluoroscopic videos was 0.24 mSv (PCXMC software, STUK, Helsinki, Finland).

### Image analysis

Manual image analysis was performed using a MATLAB-based program [16]. Twenty-two osseous points were marked in accordance with previously validated and published procedures [8, 10, 16, 17]. “The marking points were 2 anterior and 2 posterior external markers for occiput (C0), 2 points at the centers of the medullary cavities of anterior and posterior arcs of atlas (C1), 2 inferior corners of axis (C2), 2 superior corners of the seventh vertebra (C7), and the anterior and posterior corners of the superior and inferior endplates of the third to the sixth vertebrae (C3-C6)” [13].

The MATLAB program calculated joint rotation in degrees using the vertebral midplane with respect to the horizontal plane, calculating the joint midline position from two neighboring mid-planes [8, 10, 11, 16, 17]. Positive degrees indicate joint motion in extension and negative degrees indicate joint motion in flexion; either motion direction could be anti-directional with respect to the pro-directional neck motion.

### Statistical analysis

Two-hundred and thirty-one joints were included in the data set, each joint performed one flexion motion and one extension motion. The initial analysis was of all joints. The secondary stage excluded joints with anti-directional end-range (12 joints for flexion and 14 for extension) in order to focus on pro-directional surplus joint motion, as the primary aim of this study was to provide a quantitative assessment of the difference between maximum joint motion and joint end-range in healthy subjects. Tertiary stage analysis assessed surplus joint motion in quartiles of the associated end-range joint motions, with the smallest end-ranges in the first quartile and the largest in the fourth. This stage involved the analysis of 113 Type S joints for flexion and 109 Type S joints for extension. The surplus joint motion (marked with green in Fig. 1) was expressed in degrees and percentages of joint end-range motion (marked with red in Fig. 1). The motion and percentages of pro-directional surplus motion were first averaged across quartiles and then averaged across joints. The quartile data was further divided into upper cervical joints (C0 and C3) and lower cervical joints (C3 and C7). The cervical spine was divided in this way due to earlier findings from the research group. Wang et al. reported that the two regions behave differently, perhaps as a result of the anatomical differences of these regions [10].

Mann-Whitney U tests were performed in order to compare the pro-directional end-range joint motion of type C and type S. Type C is illustrated in purple in Fig. 1. Statistical analysis was performed on eleven joints after the exclusion of joint sample sizes of less than seven. Three joints were excluded due to a low sample size (for flexion C1/C2 and C6/C7 and for extension C0/C1). Minimum sample size for statistical tests was set at *n* = 7.

The final stage re-introduced type A, 12 joints for flexion and 14 for extension, (marked with blue in Fig. 1) and looked at the frequency and contribution to motion for each of the three joint types.

Data was tested for Normality using Shapiro-Wilk and Kolmogorov-Smirnov test in SPSS (IBM Statistics 26). Comparisons of joint motion were performed with independent sample t-tests and Mann Whitney U tests. Significance was accepted at *p* < 0.05. Data was presented as mean ± SD and in percentages of end-range joint motion. The average, standard deviation and range were calculated for individual joints and across joints.

## Results

Thirty-three subjects participated in the study, of which 12 were female. The demographics for the subjects can be found in Table 1.

**Table 1:** Subject Demographics. Demographic characteristics of the 33 subjects included in this study. Age, height, weight and body mass index (BMI) are shown as a mean ± SD

Demographics	Males (21)	Females (12)
Age (years)	27.0 ± 5.3	23.8 ± 3.0
Height (cm)	179.0 ± 8.4	164.4 ± 7.9
Weight (kg)	73.7 ± 9.7	61.2 ± 12.6
BMI (kg/m2)	22.9 ± 1.8	22.5 ± 2.9

Six joints (C2/C3, C3/C4 and C4/C5 for flexion and C1/C2, C3/C4 and C6/C7 for extension) out of the eleven joints (C0/C1, C2/C3, C3/C4, C4/C5 and C5/C6 for flexion and C1/C2, C2/C3, C3/C4, C4/C5, C5/C6, C6/C7 for extension) showed significantly larger motion (*p* < 0.05) in degrees for type C compared to type S (Table 2). Joint motion in degrees, was for all eleven joints, numerically larger for type C than for type S.

**Table 2:** Comparisons between type C and type S. The Mann-Whitney U comparisons of pro-directional end-range motion between type C and type S. Motion direction and joints for comparison are shown in rows one and two, rows three and four show the joint motion as a mean ± SD of type C and type S. ^a^No data available, insufficient sample size of one group

**Flexion**							
**Joints**	**C0/C1**	**C1/C2**^**a**^	**C2/C3**	**C3/C4**	**C4/C5**	**C5/C6**	**C6/C7**^**a**^
**Type C**	−4.2° (4.0°)		−5.1° (1.7°)	−9.5° (2.4°)	−11.0° (4.0°)	− 11.9° (3.9°)	
**Type S**	−2.8° (2.1°)		−4.9° (3.6°)	−5.4° (3.0°)	−6.7° (4.1°)	−9.3° (4.6°)	
**P**	0.759		0.000	0.001	0.005	0.127	
**Extension**							
**Joints**	**C0/C1**^**a**^	**C1/C2**	**C2/C3**	**C3/C4**	**C4/C5**	**C5/C6**	**C6/C7**
**Type C**		10.6° (3.2°)	6.2° (3.2°)	10.1° (3.9°)	11.1° (4.1°)	8.6° (3.9°)	7.8° (4.8°)
**Type S**		6.4° (4.3°)	5.4° (3.5°)	6.08° (3.7°)	9.3° (4.6°)	8.4° (5.0°)	3.5° (2.0°)
**P**		0.004	0.507	0.013	0.224	0.709	0.013

### Joint motion – flexion

Of the 231 joints included in the study for flexion motion, 106 joints (45.9%) were type C, 113 joints (48.9%) were type S, and 12 (5.2%) were type A.

The average pro-directional surplus joint motion was 2.41° ± 2.12° and range (0.07° -14.23°). Average surplus joint motion in the upper cervical (C0-C3) region, 3.07° ± 2.46° range (0.07°- .23°), was greater than in the lower cervical region (C3-C7), 1.60° ± 1.22° range (0.10° – 5.01°). The pro-directional surplus flexion motion is presented in Table 3.

**Table 3:** Pro-directional surplus joint motion. Pro-directional surplus motion, SD and range in degrees for flexion and extension joint motion (C0/C1 to C6/C7). No significant differences were found between flexion and extension surplus motion

	C0/C1	C1/C2	C2/C3	C3/C4	C4/C5	C5/C6	C6/C7
**Average Flexion**	2.36°	3.92°	2.71°	1.82°	1.47°	1.40°	1.48°
**SD Flexion**	3.20°	2.37°	1.65°	1.03°	1.18°	1.35°	2.00°
**Flexion Range**	0.07°-14.23°	0.26°-7.33°	0.07°-5.41°	0.19°-3.62°	0.18°-4.27°	0.10°-4.33°	0.38°-5.01°
**Average Extension**	1.99°	3.38°	2.57°	1.45°	1.08°	1.78°	1.34°
**SD Extension**	1.42°	1.95°	1.97°	1.18°	1.15°	1.70°	0.96°
**Extension Range**	0.02°-4.5°	0.05°-6.97°	0.57°-6.77°	0.20°-5.75°	0.06°-4.57°	0.04°-4.56°	0.08°-3.42°

### Quartiles of surplus flexion motion

The pro-directional surplus flexion joint motion was divided into quartiles of the associated end-range joint motion, with the smallest end-ranges in the first quartile and the largest in the fourth. Flexion motion surplus to end-range was demonstrated by 113 joints.

The quartile with the smallest end-ranges had an average pro-directional surplus joint motion of 2.79°, which was 152.0% of the associated end-range joint motion. The quartile with the largest end-ranges had an average pro-directional surplus joint motion of 1.94°, which was 24.3% of the averaged associated end-range joint motion.

Percentages of average pro-directional surplus flexion joint motion ranged from 21.2 to 359.4% in the upper cervical quartiles and from 0.8 to 94.0% in the lower cervical quartiles. In flexion, the upper cervical quartiles ranged from 1.18° to 4.36° and from 0.12° to 5.46° in the lower cervical quartiles. Average surplus joint motion as a percentage of end-range joint motion decreased with an increase in end-range joint motion. However, there was no clear pattern of data distribution for surplus motion in degrees (Table 4).

**Table 4:** Joint motion surplus to end-range in quartiles. The quartiles of pro-directional surplus flexion and extension motion for joints C0/C1 to C6/C7. The motions were divided into quartiles of end-range motion, with the smallest end-range motion in quartile 1 and the largest end-range motion in quartile 4. Each quartile shows the pro-directional surplus motion in degrees and as a percentage of the associated end-range motion. The first column from the left shows quartiles, the second column shows motion direction, degrees and percentages and the next seven columns show joints C0/C1, C1/C2, C2/C3, C3/C4, C4/C5, C5/C6 and C6/C7, the last two columns show upper cervical joints C0/C3 and lower cervical joints C3/C7. The table is divided in two, with the upper half showing flexion motion and a lower half showing extension motion. The number of joints in each quartile is given in the bottom row of each half. The calculations for flexion of C5/C6 and C6/C7 included only 8 and 5 joints respectively. The smallest number of joint observations in the extension table was eight, seen at C0/C1

**Quartile**	**Flexion**	**C0C1**	**C1C2**	**C2C3**	**C3C4**	**C4C5**	**C5C6**	**C6C7**	**C0C3**	**C3C7**
1	Degrees	2.20°	4.36°	4.13°	1.96°	2.50°	1.68°	2.73°	3.56°	2.22°
1	Percent	200.70%	204.06%	359.44%	92.64%	93.97%	37.43%	75.65%	254.73%	74.92%
2	Degrees	1.52°	2.92°	3.76°	2.26°	0.57°	2.56°	0.44°	2.73°	1.45°
2	Percent	78.63%	62.41%	100.75%	50.42%	11.93%	38.11%	7.87%	80.60%	27.08%
3	Degrees	1.91°	4.35°	1.18°	1.44°	1.49°	1.25°	0.38°	2.48°	1.14°
3	Percent	69.01%	61.71%	21.16%	25.61%	20.38%	11.61%	4.04%	50.62%	15.41%
4	Degrees	3.86°	3.33°	2.41°	1.63°	1.14°	0.12°	1.12°	3.20°	1.0°
4	Percent	76.01%	31.31%	26.26%	17.10%	9.59%	0.77%	8.85%	44.52%	9.08%
Number of joints		18	22	22	20	18	8	5	62	51
**Quartile**	**Extension**	**C0C1**	**C1C2**	**C2C3**	**C3C4**	**C4C5**	**C5C6**	**C6C7**	**C0C3**	**C3C7**
1	Degrees	2.38°	4.53°	3.37°	1.51°	1.25°	0.99°	1.49°	3.43°	1.31°
1	Percent	184.00%	171.05%	232.31%	89.68%	33.89%	29.69%	137.52%	195.85%	72.69%
2	Degrees	1.92°	3.26°	1.73°	2.02°	1.73°	3.20°	1.20°	2.30°	2.04°
2	Percent	35.05%	75.04%	48.18%	40.39%	25.11%	53.14%	36.12%	52.75%	38.69%
3	Degrees	0.96°	2.64°	2.88°	1.61°	0.77°	2.39°	1.22°	2.16°	1.45°
3	Percent	8.55%	40.16%	46.93%	22.44%	7.31%	28.85%	27.24%	31.88%	21.46%
4	Degrees	2.68°	3.11°	1.28°	0.66°	0.33°	0.52°	1.42°	2.36°	0.74°
4	Percent	12.39%	25.81%	13.19%	6.35%	2.24%	3.27%	24.42	17.13%	9.07%
Number of joints		8	20	16	16	16	16	17	44	65

### Joint motion- extension

Of the 231 joints included in the study for extension motion, 108 joints (46.8%) were type C, 109 joints (47.2%) were type S and 14 joints (6.1%) were type A. The average pro-directional surplus joint motion was 2.02° ± 1.70° range (0.04° – 6.97°).

As with flexion the average pro-directional surplus joint motion in the upper cervical region 2.84° ± 1.91° range (0.05°-6.97°) was numerically larger than in the lower cervical region 1.42° ± 1.27° range (0.04֯-4.75°). The pro-directional surplus extension motion is presented in Table 3.

### Quartiles of surplus extension motion

The pro-directional surplus extension joint motion was divided into quartiles of the associated end-range joint motion, smallest end-ranges in the first quartile, largest in the fourth. The quartile with the smallest end-ranges demonstrated an average pro-directional surplus joint motion of 2.21°, which was 87.5% of the associated end-range joint motion. The quartile with the largest end-ranges demonstrated an average pro-directional surplus joint motion of 1.43°, which was 10.3% of the associated end-range joint motion. The quartile percentage range across the upper cervical joints was 8.6 to 232.3% and across the lower cervical joints was 2.2 to 137.5% (Table 4). Surplus extension motion for the upper cervical joints ranged between 0.96° and 4.53°, the range for the lower cervical joints was between 0.33° and 3.20°. Average surplus joint motion, both in degrees, and as a percentage of end range joint motion decreased as end-ranges increased.

### Anti-directional end-range motion

Anti-directional end-range motion was demonstrated by 5.2% of cervical joints during flexion motion and 6.1% of cervical joints during extension motion. Of those joints, 0.9% during flexion and 2.2% during extension moved anti-directionally from the outset and never passed upright pro-directionally.

For flexion, the average anti-directional motion was 2.33° ± 2.53° with a range of 0.03° to 18.50°. For extension, the average anti-directional motion was 2.24° ± 1.71° with a range of 0.09° to 8.73°. Anti-directional end-range motion was found predominantly in the upper cervical region. Eleven of the 12 joints exhibiting anti-directional end-range in flexion, and 9 of the 14 in extension were found at the C0/C1-C2/C3 levels (Table 5).

**Table 5:** Anti-directional joint motion. Table 5 shows anti-directional end-range motion and anti-directional surplus motion for flexion and extension

	Anti-directional flexion				Anti-directional extension			
	End-range		Surplus		End-range		Surplus	
Joints	Count	Mean ± SD	Count	Mean ± SD	Count	Mean ± SD	Count	Mean ± SD
**C0/C1**	5	2.93 ± (1.53)	13	2.18 ± (1.93)	1	2.72	12	2.15 ± (1.42)
**C1/C2**	5	5.97 ± (2.68)	14	3.11 ± (4.67)	4	3.72 ± (0.98)	8	2.47 ± (1.73)
**C2/C3**	1	2.16	14	2.08 ± (1.71)	4	1.84 ± (1.48)	15	2.83 ± (1.82)
**C3/C4**	–	–	13	1.26 ± (0.92)	1	0.3	14	1.78 ± (1.30)
**C4/C5**	–	–	9	1.20 ± (1.07)	–	–	10	1.65 ± (1.23)
**C5/C6**	–	–	16	1.27 ± (1.03)	–	–	15	1.23 ± (0.87)
**C6/C7**	1	0.32	18	2.04 ± 1.75)	4	–	21	1.80 ± (1.38)

On average 1 out of every 18 joints produced anti-directional end-range.

### Frequency of surplus motion

The end-range position was passed pro-directionally in flexion by 54.1% and in extension by 52.3% of the 231 joints. Similarly, the upright position was passed anti-directionally by 52.3% of joints in flexion and 53.3% of all 231 joints in extension. Both the upright position and the end-range position were passed by 22.9% of all 231 joints during flexion and extension.

### Average joint motion

The average contribution to cervical ROM (C0 to C7) was in fact larger for type C than for type S, with contributions 60.23° and 67.86° for type C and 42.22° and 49.05° for type S, for flexion and extension respectively.

**Fig. 3:**
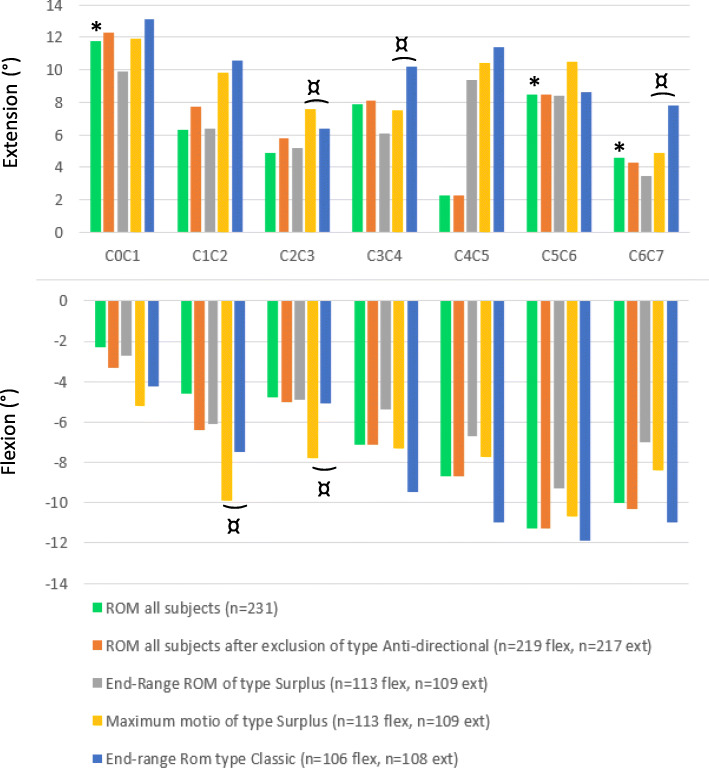
Average flexion and extension joint ROM. * Indicates a significant difference when comparing average flexion and extension. ¤ indicates a significant difference in the end-range motion of type S and type C

The average joint motion from upright to end-range for all 33 subjects before and after exclusion of type A joints is presented in Fig. 3. No type A joints were found at the C4/C5 and C5/C6 level. However, type A motion excursions were found at the remaining cervical joint levels. The figure illustrates the difference between end-range and maximum motion of type S across joints. The maximum demonstrated joint motion across subjects for each joint measured in this study is presented in Table 6.

**Table 6:** Maximum demonstrated motion. The maximum demonstrated flexion and extension motion in degrees

	C0C1	C1C2	C2C3	C3C4	C4C5	C5C6	C6C7
**Flexion**	17.7	19.4	15.6	12.8	20.4	18.6	18.3
**Extension**	26.7	21.2	14.6	18.0	17.8	19.4	16.3

Assessment of average flexion and extension joint motion of all joints using t-tests showed a significant difference (*P* < 0.0001) for C0/C1, where 11.85° of extension was considerably larger than − 2.33° of flexion. In contrast flexion, at − 11.26° for C5/C6 and − 10.01° for C6/C7 was significantly larger than extension, at 8.51° for C5/C6 and 4.56°, for C6/C7, (*P* < 0.05) and (*P* < 0.0001) respectively.

The end-range joint motions of type C and type S were significantly different for C2/C3, C3/C4 and C4/C5 (*P* < 0.01) in flexion and C1/C2, C3/C4 and C6/C7 (*P* < 0.05) in extension (Table 2).

## Discussion

This study changes our understanding of cervical motion by demonstrating that a little under half of the cervical joints (48.1%) produced pro-directional surplus motion with an average of approximately 2°. Surplus motion should not be considered abnormal as 113 out of 219 joints in flexion and 109 out of 217 joints for extension demonstrated joint motion surplus to end-range.

Approximately 1/5 of all joints demonstrated both pro-directional and anti-directional surplus motion, passing upright and end-range positions with similar frequency. Those joints that did not produce pro-directional surplus joint motion (type C) comprised 46.3% of the total joints.

Interestingly 5.7% of all joints displayed anti-directional end-range joint motion (type A).

Type A joints were found predominantly in the upper cervical region, with only a few in the mid and lower cervical regions, suggesting that the anatomical structure of the vertebrae may influence the prevalence of this motion [9, 18]. The finding that joints can complete their motion in opposition to the direction of head motion is not unexpected. Previous documentation that large proportions of anti-directional cervical flexion and extension motions were normal in healthy subjects, gave some indication of this possibility [10, 13].

### Surplus motion

This study suggests that it may be possible to use the average pro-directional surplus joint motion as a percentage of end-range ROM as an indicator for the reliability of end-range motion to predict the maximum joint motion. Analysis of the quartiles of surplus motion demonstrated a clear pattern for both flexion and extension. Surplus joint motion as a percentage of end-range joint motion decreased with an increase in end-range motion.

As small end-ranges are associated with large percentages of surplus motion, using end-range in these situations to predict a joint’s maximum motion should be done with caution. Conversely, it could be argued that large end-ranges can be more readily utilised as a predictor for maximum joint motion due to their association with small percentages of surplus motion. This does however question the reliability of flexion-extension X-rays as an accurate indicator for a joints maximum motion.

### Maximum demonstrated joint motion

The data suggests that the end-range motion does not reflect the maximum possible motion for an individual joint. This is especially clear for C0/C1 during flexion, where the average ROM was 2.33° and the average pro-directional surplus motion was 2.36° with a range up to 14.23°. The upper cervical joint appeared to flex in the beginning of the flexion motion excursion, but to move anti-directionally later in the motion, towards a lesser degree of flexion. The small average motion of C0/C1 does not reflect the maximum motion capacity of C0/C1 during flexion. This is illustrated by the maximum joint motion of type S for C0/C1 (Fig. 3) and the large range for pro-directional surplus motion for C0/C1, shown in Table 3. The maximum possible motion of healthy cervical joints is therefore unknown. It is not clear if the maximum measured motion found for all single joints in this study reflects the maximum possible motion capacity of healthy cervical joints, but we consider this unlikely.

The average cervical ROM measured between upright and end-range in this study was similar to previous reports despite differences in the methodology [3, 8, 9].

Cervical joint motion between upright and end-range positions has previously been assessed by Wu et al. using video fluoroscopy. In this case motion was assessed in ranges of one third and the C0/C1 joint was omitted from the study. The current study showed that end-range flexion and end-range extension joint motion were significantly different for C0/C1, C5/C6 and C6/C7. By assessing ROM in 10% epochs, this study aimed to give a more detailed picture of the joint motion pattern. The C0/C1 joint was also included in this study as we know it to be important in its contribution to cervical spine motion [15].

The cervical flexion motion of C0/C1 (2.3°) demonstrated the smallest average joint motion found in this study. No previous studies have reported the amount of motion found between upright and end-range flexion for C0/C1. One study reported end-range flexion to end-range extension motion for C0/C1, and the combined flexion and extension motion of that study was comparable to the findings of this study [8].

### Clinical implications

The results indicate that the end-range motion seen on flexion-extension X-rays may not be reliable for the diagnosis of reduced joint motion, as joints with small end-range motion were associated with large surplus joint motion percentages. In contrast, cervical joints with large end-range motion were associated with small percentages of surplus joint motion, consequently offering a more reliable prediction of the maximum motion of a joint. It is a reasonable consideration that in order for the joints of the cervical spine to produce multiplane motion, a joint’s motion capacity cannot be expended by motion in a single plane. However, it is clear that in most clinical interpretations of neck motion the concept of surplus motion is not applied.

Orthopedic surgeons use the terminology compensation for additional joint motion found in joints adjacent to a surgical fusion. Several biomechanical studies have documented a mechanism by which adjacent unfused levels compensate for the loss of cervical range of motion (ROM) in fused levels [19]. The compensation is perceived as a new ability for further cervical single joint motion; however, the compensation may be pre-existing surplus motion of the adjacent joints. This clinical implication may raise the question: is the success of surgical fusion dependent upon pre-surgical surplus motion in the adjacent joints?

Chiropractors have previously used the term para-physiological space to explain the motion which allows an adjustment to occur when a cervical joint is brought to tension.

However, it is possible that the para-physiological space may simply be the surplus motion of the cervical joints. It would seem that we cannot fully understand cervical motion during a physical examination, the fixation or the manipulation without first having a better understanding of surplus joint motion. The complexity of joint motion has been demonstrated in recent research [10, 13, 17, 20–22].

### Study limitations

Quantification and analysis of video-fluoroscopy has some limitations. The largest confounder is the measurement error; however, the experimental procedures and reproducibility of image analysis have previously been published [16]. High reliability of the vertebral marking procedure has been established and high ICCs have been documented in previous studies [10, 16].

Likewise, repeatability of the joint motion angle has previously been published [13]. Although Wang et al. [13] demonstrated that cervical joints accurately repeat their motion; it must be acknowledged that they were not investigating surplus motion, but joint motion angles.

It may be considered a limitation of the study that data was taken from a single motion excursion, rather than taking an average of multiple excursions, however this decision was made in order to reduce radiation exposure to subjects.

The study group was primarily younger adult males and females, which raises the question: are the results applicable to an older population? Other demographic or anatomical stratification for sex, age, height, weight, posture, and type of neck: long, thin, short and amount of adipose tissue, may also influence the cervical ROM and the study results, potentially limiting their application.

Variations in the curvature of the neck, were not considered central to the investigation as all patients were deemed healthy and screened for previous trauma, disease processes or episodes of previous cervical pain. Additionally, cervical ROM in this study was similar to the results of previous studies [3, 8, 9].

It is recognised in this study that surplus joint motion can be both pro-directional and anti-directional and that some joints produce surplus motions in both directions. For the purpose of clarity, and because the focus of this paper is maximum pro-directional joint motion, joint classification in this study is based on end-range. While type C joints in this study do not demonstrate pro-directional surplus motion, a proportion of these joints is very likely to produce anti-direction surplus motion. Likewise, a proportion of type S joints will likely produce anti-directional surplus motion. It is also of note that the variability in joint motion will influence how joints are grouped (type C, S and A) from motion to motion.

It could also be argued that the study is limited by the choice only to include flexion and extension, as this does not allow us to investigate the full dynamic capability of the joints in multiple planes. However, there must be consideration given to the level of radiation exposure healthy subjects are subjected to.

#### Future studies

Future studies may look at the effect of variations in the cervical lordosis and age, among other demographic variations, on the prevalence and distribution of surplus joint motion in healthy adults.

The quantification of surplus motion will provide reference values against which symptomatic patient data can be compared. Future investigations into the effect of pain on surplus motion would be beneficial, in order to establish the diagnostic utility of surplus joint motion. Studies of pain effects on joint motion have documented that both experimental and recurrent neck pain altered anti-directional motion patterns in the cervical spine [20–22]. Lastly a more detailed classification of joint types may be of interest, addressing the prevalence of anti-directional surplus motion.

## Conclusion

This is the first study to categorise joints by type of motion. Type S constituted approximately half of the joints analysed in this study. Therefore, end-range motion cannot be assumed to be a demonstration of a joint’s maximum motion. This brings into question the reliability of flexion/extension X-rays as a measure of the total motion capacity of the cervical spine. The traditional view of joint motion seems to describe the motion pattern of a type C joint. Only half the joints represented in this study produced a type C motion pattern, suggesting that the traditional view of joint motion represents an incomplete picture.

Until now the presence of surplus joint motion has been acknowledged, but never quantified, yet it is undeniably a persistent finding.

## Data Availability

The datasets generated and/or analysed during the current study are not publicly available due to data protection of participants but are available from the corresponding author on reasonable request.
